# The economic impact of alcohol consumption: a systematic review

**DOI:** 10.1186/1747-597X-4-20

**Published:** 2009-11-25

**Authors:** Montarat Thavorncharoensap, Yot Teerawattananon, Jomkwan Yothasamut, Chanida Lertpitakpong, Usa Chaikledkaew

**Affiliations:** 1 Health Intervention and Technology Assessment Program (HITAP), Ministry of Public Health, Bangkok, Thailand; 2 Department of Pharmacy, Faculty of Pharmacy, Mahidol University, Bankok, Thailand

## Abstract

**Background:**

Information on the economic impact of alcohol consumption can provide important evidence in supporting policies to reduce its associated harm. To date, several studies on the economic costs of alcohol consumption have been conducted worldwide. This study aims to review the economic impact of alcohol worldwide, summarizing the state of knowledge with regard to two elements: (1) cost components included in the estimation; (2) the methodologies employed in works conducted to date.

**Methods:**

Relevant publications concerning the societal cost of alcohol consumption published during the years 1990-2007 were identified through MEDLINE. The World Health Organization's global status report on alcohol, bibliographies and expert communications were also used to identify additional relevant studies.

**Results:**

Twenty studies met the inclusion criteria for full review while an additional two studies were considered for partial review. Most studies employed the human capital approach and estimated the gross cost of alcohol consumption. Both direct and indirect costs were taken into account in all studies while intangible costs were incorporated in only a few studies. The economic burden of alcohol in the 12 selected countries was estimated to equate to 0.45 - 5.44% of Gross Domestic Product (GDP).

**Conclusion:**

Discrepancies in the estimation method and cost components included in the analyses limit a direct comparison across studies. The findings, however, consistently confirmed that the economic burden of alcohol on society is substantial. Given the importance of this issue and the limitation in generalizing the findings across different settings, further well-designed research studies are warranted in specific countries to support the formulation of alcohol-related policies.

## Background

Alcohol exerts a substantial economic burden worldwide [1-3]. The need for estimates of the economic cost of alcohol is almost self-evident. This estimation is potentially a valuable source of information for policymakers, researchers and public health planners. Specifically, it is useful in supporting a formulation of alcohol-related policies and in planning and estimating the cost-effectiveness of policies or interventions aimed at mitigating the negative consequences of alcohol consumption. In addition, it can be used to identify information gaps, research needs and adjustments to national statistical reporting systems. Also, it increases public awareness of the economic burden alcohol has on society.

Estimating the economic costs of alcohol abuse poses a number of methodological challenges and the magnitude of the cost estimates can vary depending on the methodology employed. Two approaches, namely the prevalence and incidence approaches have been widely used in cost-of-illness studies. These two approaches are used to address different research questions. The incidence approach estimates the costs and consequences associated with new drinkers in the current and future years, while the prevalence approach estimates costs associated with past and current use in a given year.

In estimating the costs related to premature mortality, the demographic and human capital approaches are most commonly used. Each approach addresses different questions and can be considered complementary rather than contradictory. The choice of approach might also depend on the availability of information in different settings. The demographic approach compares the actual output from the population size and structure with that of a hypothetical population where no alcohol abuse occurs. In contrast, the human capital approach estimates the loss of future streams of productive capacity, expressing this at present day value by the application of an appropriate discount measure.

Economic costs associated with alcohol consumption can be estimated either in terms of gross cost or net cost. Net cost estimation takes into account the possible positive effects that could be generated from alcohol consumption. Gross cost estimation, on the other hand, estimates only the costs associated with the negative effects of alcohol consumption.

To date, several studies on the economic costs of alcohol abuse have been conducted across settings worldwide [4-20] using different methods. A systematic review of these studies is essential for all stakeholders who want to keep up with the accumulating evidence in this field. A prior review of studies on the economic cost of alcohol published between 1990 to 2004 was conducted by Baumberg [1], suggesting that the economic burden associated with alcohol at the global level ranged from $US 210 to 665 Billion in 2002. Two possible limitations of this review were identified. First, the global burden of alcohol was estimated using studies from developed countries alone. Second, the inclusion of different cost components in the estimates, which can explain major discrepancies in the individual study results, was not examined.

This study has two objectives: (i) summarize the current estimates of cost of alcohol abuse worldwide; and (ii) compare the similarities and differences in terms of cost components and methods employed in the works conducted to date.

## Methods

A systematic literature review was conducted by searching MEDLINE electronic databases to identify relevant publications between 1990-2007 concerning the economic costs or the social costs of alcohol. WHO's global status report on alcohol 2004[2], bibliographies, and expert communications were also used to identify additional further relevant studies. The literature searches were based on the combined searches of the following terms: *alcohol AND ("cost of illness" OR "social cost" OR "social costs" OR "economic cost" OR "economic costs" OR "societal cost" OR "societal costs")*. The titles and abstracts of the publications identified were assessed by two independent reviewers. If the abstracts were deemed to be relevant, full transcriptions of the papers were then obtained. The selection criteria used in this review are outlined in table 1. If several publications on the same study were identified, the original publication was selected for the review. Where the full description of the original publication could not be retrieved, another publication of the same study was selected instead. For each eligible study, the following elements were extracted by standardized data extraction forms; 1) methodological characteristics, 2) total estimated cost of alcohol, 3) cost components included in the analysis, as well as its magnitude, and 4) types of diseases included in the estimation.

**Table 1:** Inclusion and exclusion criteria

Inclusion criteria	Exclusion criteria
• Studies that consider the economic costs of alcohol or social cost of alcohol	• Non-English Language
• English language	• Conference abstract
• Published during 1990 -- 2007	• No costs quoted in the result section
	• Conducted in specific population sub-groups such as pregnant women or adolescents
	• Not enough information to identify methodologies used in the study
	• Unable to retrieve full description of the publication
	• Not an original research article (i.e. review articles, systematic review articles, and editorials)
	• Further publications of single studies

### Methodological characteristics

Methodological characteristics extracted included 1) the approaches used in the cost estimates, i.e. prevalence or incidence approach, 2) the methods used for estimating the cost of premature mortality, i.e. human capital or demographic approach, 3) the inclusion of the positive effects of alcohol drinking, i.e. using gross cost or net cost estimates, and 4) the discount rate used for adjusting future monetary values.

### Total estimated cost of alcohol

For each eligible study, the total estimated costs of alcohol and total cost estimates in term of Gross Domestic Product (GDP) or Gross National Product (GNP) were presented as originally published. To facilitate the comparison across different settings and years, total cost estimates were also presented in 2007 $US values by inflating the original cost to its 2007 value using country-specific GDP inflators[21]. The costs were then converted into US dollars using Purchasing Power Parities (PPP)[21]. To facilitate further comparison across studies in term of the magnitude of the total estimates relative to GDP, we calculated the total cost and direct cost as percentage of GDP (PPP) 2007 and cost per capita, using information from The World Economic Outlook Database by the International Monetary Fund (IMF)[21].

### Cost components included in the analysis

In this study, cost components in each identified study were classified into three main categories, namely: direct costs, indirect costs, and intangible costs. Direct costs measure the value of resources used as a consequence of alcohol abuse. The direct cost in this study was further classified into 1) health care cost, 2) research and prevention costs, 3) costs of crime and law enforcement, 4) costs of property damage or loss, 5) administration costs, 6) costs of welfare assistance or social work. (These costs did not, however, include any welfare payments), 7) costs of alcohol beverage, and 8) other costs.

In contrast, indirect costs are those for which resources are lost without a direct payment actually being made. In this study, indirect costs have been classified into five categories as follows; 1) the cost of premature mortality, 2) the cost of reduced productivity, which includes both the cost of productivity loss due to absenteeism and that when the workforce comes to work (presenteeism), 3) the cost of incarceration, 4) the cost of loss of employment or early retirement, and 5) costs associated with crime i.e. time loss for victims due to crime.

The last cost category is referred to as intangible costs, which represented pain, suffering, and the deterioration of quality of life. This type of cost, when reduced or eliminated, does not yield resources that can be made available for other uses, and is less likely to be included in cost estimations[22].

The type of alcohol attributed diseases included in the estimation can also be an important factor. In this study, diseases were classified according to the International Statistical Classification of Diseases and Related Health Problem 10^th ^Revision (ICD-10) into 10 groups as follows; A = Infectious diseases(A00-B99), B = Neoplasm(C00-D48), C = Endocrine diseases (E00-E99), D = Mental and behavioural disorders (F00-F99), E = Diseases of the nervous system(G00-G99), F = Diseases of the circulatory system (I00-I99), G = Diseases of the digestive system (K00-K93), H = Diseases of the skin (L00-L99), I = Conditions associated with pregnancy or child birth, and certain conditions in the perinatal period (O 00-O99, P00-P96), J = Injuries, poisoning, and other external causes (S00-T-98, V01-Y98).

## Results

### Literature search

The initial search strategy, conducted in August 2007, identified 318 potential relevant articles from MEDLINE databases. Of these, only 9 studies [8,9,11,12,14,16,18-20] fulfilled the eligibility criteria, as indicated in table 1. Eleven additional eligible studies were identified through expert communication and bibliographies of reviewed studies[4,6,7,10,13,15,17,23-25] Upon completion of this strategy, 20 studies[4,6-20,23-25] conducted in 12 developed countries (Australia, Canada, England and Wales, France, Germany, Japan, the Netherlands, New Zealand, Portugal, Scotland, Sweden and the United States) and 1 developing countries (Thailand) [25]were included in the full review. It should be noted that the Australian study [6] contained cost estimations for 2 different years (1988 and 1992).

**Figure 1:**
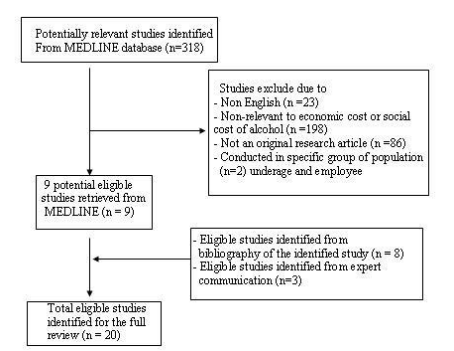
**Search and retrieval process**.

One non-English study with English summary and tables conducted in South Korea [5]and one English article with only cost summary table conducted in Finland [26]were also included in the partial review. Other 11 non-English studies conducted in Spain[27], Italy[28], Germany[29], Argentina[30], Chile[31], Norway[32], Switzerland[33], France[34,35], Slovenia[36], and Belgium[37] were also identified by expert communications but were not included in this review. Search and retrieval processes are shown in figure 1.

### General Methodological characteristics

The general characteristics of the studies included in this review have been summarized in table 2. It was found that all of the studies identified were prevalence-based studies using societal perspective. However, it should be noted that only external costs (costs that affect others than the consumers) were taken into account since private costs (costs that affect the consumers) were considered to be offset by benefit from the consumption. Nevertheless, a few studies [5-7]included private cost (i.e. cost of alcoholic beverage) in their estimations.

Regarding the inclusion of the positive effects of alcohol drinking, only three studies [6,7] from Australia estimated the economic cost of alcohol in terms of net cost, while 17 studies [4-10,13-17,19,20,23-25] calculated the gross cost. Two recent studies in Germany [12]and Sweden [11] estimated total costs in term of both net cost and gross cost and found that the net cost estimation was approximately 12.5-14.5% lower than the gross cost estimation.

When the method employed in estimating the cost of premature mortality was reviewed, it was found that three studies, all conducted in Australia[6,7], adopted the demographic approach, while the remaining 17 studies [4,5,8-12,14-20,23-25]employed the human capital approach. Of the 17 studies that employed the human capital approach [4,5,8-12,14-20,23-25], the discount ranged from 3%[11,25] to10%[8], with the most frequently cited value being 6%[9,10,14,16-20,23], complying with the 5 - 10% range recommended by WHO's guideline [22].

**Table 2:** Methodological characteristics of the 22 studies reviewed

Study	Consideration of positive effect	Approach	Discount rate
Australia 1988 [6]	Net cost	Demographic approach	N.A.
Australia 1992 [6]	Net cost	Demographic approach	N.A.
Australia 1998-9 [7]	Net cost	Demographic approach	N.A.
Canada 1992 [18]	Gross cost	Human capital approach	6%
Canada 2002 [15]	Gross cost	Human capital approach	5%
Ontario 1992 [20]	Gross cost	Human capital approach	6%
England and Wales 2001/2 [4]	Gross cost	Human capital approach	N.I
Finland 1990 [26]	N.I	N.I	N.I
France 1997 [9]	Gross cost	Human capital approach	6%
Germany 2002 [12]	Gross cost & Net cost	Human capital approach	5%
Japan 1987 [14]	Gross cost	Human capital approach	6%
The Netherlands 2001 [13]	Gross cost	N.A.	N.A.
New Zealand 1991 [8]	Gross cost	Human capital approach	5 &10%
Portugal 1995 [24]	Gross cost	Human capital approach	5%
Scotland 2001/2 [19]	Gross cost	Human capital approach	6%
Sweden 2002 [11]	Gross cost & Net cost	Human capital approach	3%
South Korea 2000 [5]	Gross cost	Human capital approach	5%
Thailand 2006 [25]	Gross cost	Human capital approach	3%
US 1985 [17]	Gross cost	Human capital approach	6%
US 1990 [16]	Gross cost	Human capital approach	6%
US 1992 [23]	Gross cost	Human capital approach	6%
US 1998 [10]	Gross cost	Human capital approach	6%

NI: Not indicated, NA: Not applicable

### Cost components included in the estimates

Table 3 gives an overview of the cost components included in all 22 reviewed studies. Both the direct costs and indirect costs were reported in all studies, while intangible costs were reported in only 5 [4,6,7,11].

**Table 3:** Cost components included in 22 studies reviewed

		A	B	C	D	E	F	G	H	I	J	K	L	M	N	O	P	Q	R	S	T	U	V
Direct cost																							
*Health care*		+	+	+	+	+	+	+	+	+	+	+	+	+	+	+	+	+	+	+	+	+	+
*Research and prevention*		-	-	-	+	+	+	-	-	+	+	+	-	-	-	+	+	-	-	+	+	+	+
*Crime and law enforcement*		+	+	+	+	+	+	+	+	+	-	+	+	+	+	+	+	-	+	+	+	+	+
*Property damage/Loss*		+	+	+	+	+	+	+	+	+	-	+	+	-	+	-	-	+	+	+	+	+	+
*Administration cost*		+	+	-	+	+	+	-	+	-	+	+	+	-	+	-	-	+	-	+	+	+	+
*Welfare assistance/social work*		-	-	-	-	-	-	-	+	-	-	+	-	-	+	+	+	-	-	-	-	-	-
*Alcohol beverage*		+	+	+	-	-	-	-	-	-	-	-	-	-	-	-	-	+	-	-	-	-	-
*Other*^*b*^		-	-	-	-	-	-	+	+	+	-	+	+	+	-	-	-	-	-	-	-	-	-
Indirect cost																							
*Premature mortality*		+	+	+	+	+	+	+	+	+	+	+	-	+	+	+	+	+	+	+	+	+	+
*Reduced productivity*		+	+	+	+	+	+	+	+	+	+	+	+	+	+	+	+	+	+	+	+	+	+
*Incarceration*		-	-	+	-	-	-	-	+	+	-	-	-	+	+	+	+	-	-	+	+	+	+
*Loss employment*		-	-	-	-	+	-	+	-	-	+	+	+	+	+	+	+	-	-	-	-	-	-
*Victim time*		-	-	-	-	-	-	+	-	-	-	-	-	-	+	-	+	-	-	+	+	+	+
Intangible cost		+	+	+	-	-	-	+	-	-	-	-	-	-	-	-	-^a^	-	-	-	-	-	-

+ = Yes, - = No,

a = the reduction in quality of life is measured but cost associated with the reduction was not reported b = Other direct costs included the cost of anticipating crime, the cost of losses from compulsory taxes, and transfer cost (i.e. disability pension, welfare payment, accident compensation, and social security payment) [8,13,14,26]

A = Australia 1988 [6], B = Australia 1992 [6], C = Australia 1998-9[7], D = Canada 1992[18], E = Canada 2002[15], F = Ontario 1992[20], G = England and Wales 2001/2 [4], H = Finland 1990 [26], I = France 1997[9], J = Germany 2002[12], K = Japan 1987[14], L = The Netherlands 1991[13], M = New Zealand 1991[8], N = Portugal 1995[24], O = Scotland 2001/2[19], P = Sweden 2002[11], Q = South Korea 2000[5], R = Thailand 2006[25], S = US 1985 [17], T = US 1990 [16], U = US 1992[23], V = US 1998 [10]

As for direct costs, health care costs and the cost of crime and law enforcement were identified in all but 2 of the 22 studies, conducted in Korea [5]and Germany[12], in which the cost of crime and law enforcement was not taken into account. The cost of property damage or loss was taken into account in all studies except those from New Zealand[8], Scotland[19], Sweden [11]and Germany[12]. The cost of research and prevention, and administrative costs were identified in 12[9-12,14-20,23] and 15 studies[5,6,10,12-18,20,23,24,26], respectively. Very few studies included the cost of alcohol beverages [5-7]or welfare assistance/social service costs [11,14,19,24,26].

Regarding indirect costs, the costs of reduced productivity were calculated in all 22 studies. All of the studies, with one exception[13], also incorporated the cost of premature mortality into their estimations. The cost of productivity loss due to incarceration and time loss for victims of crime was calculated in 10[7-11,16,17,23,24,26], and 7 studies[4,10,11,16,17,23,24], respectively.

Only 5 studies included intangible costs in their estimation[4,6,7,11] Most of these studies employed the willingness-to-pay method[38] in estimating intangible costs. The value of life was estimated at 2,000,000 Australian dollars (in 1996) in Australia[7]. Contrastingly, in the study conducted in England and Wales[4], the intrinsic value lost for a fatal causality was estimated to be £783,000 (in 2002). It should be noted that in the Swedish study[11] the intangible cost was measured in terms of Quality-Adjusted Life Years (QALYs) lost. In this study, the QALY lost due to mortality for consumers, friends, relatives and victims of crime was 121,791 and 145,356 QALY in net and gross estimation, respectively.

Diseases attributable to alcohol included in the estimation are displayed in table 4. The review showed some variations in the number of disease groups included in each study. As shown in table 4, only 5 studies included infectious diseases in their estimations, while neoplasm, mental disorders, diseases of the circulatory system, diseases of the digestive system, and injury, poisoning, and external causes were considered in all studies. Also, it should be noted that one study [13]included only wholly alcohol-attributable diseases.

**Table 4:** Classification of diseases included in the estimates of the 22 studies reviewed

Study		Disease class									
	
		A	B	C	D	E	F	G	H	I	J
Australia 1988 [6]		N.I.									
Australia 1992 [6]		N.I.									
Australia 1998-9 [7]		-	+	-	+	+	+	+	+	+	+
Canada 1992 [18]		N.I.									
Canada 2002 [15]		-	+	+	+	+	+	+	+	+	+
Ontario 1992 [20]		-	+	-	+	+	+	+	+	+	+
England and Wales 2001/2 [4]		-	+	-	+	+	+	+	-	+	+
Finland 1990 [26]		N.I.									
France 1997 [9]		N.I.									
Germany 2002 [12]		-	+	+	+	+	+	+	+	+	+
Japan 1987 [14]		+	+	-	+	-	+	+	-	-	+
The Netherlands 2001[13]		-	-	-	+	-	-	+	-	-	+
New Zealand 1991 [8]		-	+	-	+	+	-	+	-	+	+
Portugal 1995 [24]		-	+	-	+	+	+	+	+	-	+
Scotland 2001/2 [19]		-	+	-	-	+	+	+	-	-	+
Sweden 2002 [11]		-	+	+	+	+	+	+	+	+	+
South Korea 2000 [5]		-	+	-	+	+	+	+	+	+	+
Thailand 2006 [25]		+	+	+	+	+	+	+	+	+	+
US 1985 [17]		N.I.									
US 1990 [16]		N.I.									
US 1992 [23]		+	+	+	+	+	+	+	-	+	+
US 1998 [10]		+	+	+	+	+	+	+	-	+	+

NI: Not indicated, NA: Not applicable, * based on ICD-10th Classification

A = Infectious diseases, B = Neoplasm, C = Endocrine diseases, D = Mental and behavioural disorders, E = Diseases of the nervous system, F = Diseases of the circulatory system, G = Diseases of the digestive system, H = Diseases of the skin, I = Conditions associated with pregnancy or child birth, and certain conditions in the perinatal period, J = Injuries, poisoning, and other external causes

### Estimated costs of alcohol

Cost estimations from each study are presented in table 5 in terms of total cost in the study year, cost as a percentage of GDP or GNP, and the share of the direct cost, indirect cost, and intangible cost, respectively. The share of the direct costs represented from 4% [25]to 52% [13]of the total cost, while the share of the indirect cost accounted for 23%[7] to 96% [25] of the total cost. Where intangible cost was considered[4,6,7,11], its share ranged from 21%[6] to 27%[7] of the total cost. When comparing across cost categories, it was found that indirect costs represented the largest proportion of the total cost in 16 of the 22 studies [5,8-12,14,16-20,23-26], as shown in table 5.

**Table 5:** Total cost and share of direct cost, indirect cost and intangible cost

Country/ Study Year	Cost as % GDP (GNP)+	Total cost in study year (Billion)^a^	Direct cost (%)	Indirect cost (%)	Intangible cost (%)
Australia 1988 [6]	N.I.	AUS$4.00	48%	31%	21%
Australia 1992 [6]	N.I.	AUS$4.5	45%	33%	21%
Australia 1998-9 [7]	1.98^b^	AUS$7.56	50%	23%	27%
Canada 1992 [18]	1.09	CAN$7.52	45%	55%	N.A.
Canada 2002 [15]	1.1-2.7^c^	CAN$14.55	51%	49%	N.A.
Ontario 1992 [20]	1.02^d^	US$2.26	44%	56%	N.A.
England and Wales 2001/2 [4]	N.I.	£18.52	38%	37%	25%
Finland 1990 [26]	(3.5)	FMK 17.31	19%	81%	N.A.
France 1997 [9]	1.42	FF 115.42	50%	50%	N.A.
Germany 2002 [12]	0.93-1.16^e^	Euro 19.56 - 24.40^e^	35%^f^	65%^f^	N.A.
Japan 1987 [14]	(1.9)	¥6,637.60	20%	80%	N.A.
The Netherlands 2001 [13]	N.I.	EUR 2.58	52%	48%	N.A.
New Zealand 1991 [8]	(1.5-5.7)^g^	NZ$1.04-3.98	15-33%	67-85%	N.A.
Portugal 1995 [24]	0.6	Euro 0.43	25%	75%	N.A.
Scotland 2001/2 [19]	N.I.	£1.07	42%	58%	N.A.
Sweden 2002 [11]	0.9-1.3^e^	SEK 20.33-29.38^e^	37%-49%^e^	51%-63%^e^	0%^h^
South Korea 2000 [5]	2.86	WON 14,935.2	28%	72%	N.A.
Thailand 2006 [25]	1.99	Baht 156.11	4%	96%	N.A.
US 1985 [17]	1.66	US$70.34	22%	78%	N.A.
US 1990 [16]	N.I.	US$98.62	23%	77%	N.A.
US 1992 [23]	N.I.	US$148.02	28%	72%	N.A.
US 1998 [10]	N.I.	US$184.64	27%	73%	N.A.

NI: Not indicated, NA: Not applicable, a: may not equal the cost quoted in the original study due to round up, b: presented in 1988 value, c: did not include intangible costs and productivity losses in non- labour force, d: % range of % GDP in provinces, e: % of Ontario GDP, f: net cost and gross cost, g; presented in terms of lower bound and upper bound, h: in gross cost estimation, i: measured in terms of Quality-adjusted life year (QALY) loss, +: as quoted in the original study.

### Direct cost

When looking at each of the direct cost components as shown in table 6, there was no general agreement across studies on what constitutes their largest share. For example, health care costs, which were included in all studies, made up 90.4% of the total direct cost in Japan[14], but was only 6 to 7% of the total direct cost in Australia[6,7]. Besides the differences due to country-specific factors, this could also be explained by the fact that net cost instead of the gross cost was estimated in these studies[6,7]. Similarly, law enforcement and criminal justice costs accounted for 68 to 80% in New Zealand[8], but less than 1% of the total direct cost in both Japan [14] and France[9].

**Table 6:** Share of each component included in the direct cost and indirect cost

Country/study Year	Total direct cost				Total indirect cost		
	
	Health care cost (%)	Crime and laws enforcement (%)	Property damage/loss (%)	Other direct cost (%)	Reduced productivi-ty (%)	Premature Mortality (%)	Other indirect cost (%)
Australia 1988 [6]	6.9	3.2	24.5	65.4	14.5	85.5	N.A.
Australia 1992 [6]	7.2	3.0	23.7	66.1	13.9	86.1	N.A.
Australia 1998-9 [7]	6.0	29.3	33.8	30.9	3.2	84.6	12.2
Canada 1992 [18]	38.4	40.1	15.3	6.2	33.8	66.2	N.A.
Canada 2002 [15]	44.5	41.4	12.3	1.8	0.6	13.0	86.4
Ontario 1992 [20]	35.6	42.1	16.0	6.3	37.8	62.2	N.A.
England and Wales 2001/2 [4]	22.7	24.2	33.3^a^	19.8	37.8	33.3	28.9
Finland 1990 [26]	28.0	16.6	13.5	41.9	4.2	95.8	N.A.
France 1997 [9]	31.8	0.6	40.0^b^	27.6	6.7	92.4	0.9
Germany 2002 [12]	83.7	N.A.	N.A.	16.3	17	68.8	14.2
Japan 1987 [14]	90.4	0.02	0.3	9.3	79.7	17.3	3.0
The Netherlands 2001[13]	13.5	8.1	54.0^c^	24.4	19.7	N.A.	N.A.
New Zealand 1991 [8]	11.4-20.6	70.9-79.5	N.A.	17.7	56.2	3.3	80.3
Portugal 1995 [24]	30.4	17.4	52.2	0	76.7	23.3	N.A.
Scotland 2001/2 [19]	20.9	59.6	N.A.	19.5	19.2	67.3	13.5
Sweden 2002 [11]	22-30	26-28	N.A.	44-49	33.1 - 44.6	29.4-46.3	20.6 - 29.1
South Korea 2000 [5]	21.7	N.A.	5.8	72.5	58.4	41.6	N.A.
Thailand 2006 [25]	84.3	3.7	12.0	N.A.	30.4	70	N.A.
US 1985 [17]	43.1^d^	26.9	19.2	10.8	50.2	44.0	5.8
US 1990 [16]	45.6^d^	25.2	19.6	9.6	48.5	44.5	7
US 1992 [23]	40.6^d^	15.4	37.1	6.9	64.7	29.3	6
US 1998 [10]	46.5^d^	12.5	34.3	6.6	64.4	28.1	7.5

NA: Not applicable, a: this figure also included cost of victim of crime support, b: this figure included all road accident expenditures of insurance companies, which covered more than cost of property damage, c: this figure also included treatment cost related to accidents, material cost, and cost of productivity losses due to accidents, d: not included healthcare cost for fetal alcohol syndrome

In the 18 studies [4-7,9,10,13-18,20,23-26] where the cost of property damage or loss was taken into account, its share ranged from 0.3% [14] to 54%[8]. In addition, two studies [4,11] included the cost of stolen property in their estimations. In the Netherlands[13], France[9], and England and Wales[4], where the costs associated with property damage accounted for the major share of the total direct cost, the estimates included the cost of victim support, the cost of medical expenses, production losses, and the cost of materials associated with road traffic accidents.

Where the cost of research and prevention and cost of administration were taken into account, its share was found to be less than 8% of the total direct cost. On the other hand, cost of welfare assistance or social work ranged widely from 0.04% in Japan[14] to about 40% of the total direct cost in Sweden[19]. When the cost of alcohol beverage was taken into account [5-7], this component represented a sizable amount, ranging from 33%-63% to 75% of the total direct cost in Australia [6,7]and South Korea [5], respectively. Regarding other direct costs, it was found that transfer costs including disability pensions, welfare payments, social security payments, and accident compensation were incorporated in several studies [8,13,14,26].

### Indirect cost

For the components of indirect cost, as shown in table 6, the cost associated with premature mortality played the largest part in contributing to the total indirect costs in just over half of the studies[6,7,9,11,12,18-20,25,26]. In the remaining 9 studies[4,5,8,10,14,16,17,23,24], the cost of reduced productivity accounted for the highest proportion of the total indirect cost. As for the cost of reduced productivity, seven studies [8,13,14,16-18,20] took into account both costs associated with absenteeism and situations where the workforce came to work, albeit with compromised productivity, with the impairment rate ranging from 20 to 25%. The cost of productivity losses due to incarceration and being a victim of crime was found to be relatively small, ranging from less than 1% to 17% of the total indirect cost.

Table 7 presents total costs in PPP US$ 2007, costs as % of GDP 2007, costs per capita, and total direct costs as % GDP in the 12 selected countries[5,7-15,24,25]. The studies selected in this table were the most recent for each country. As shown in the table, total cost as % GDP ranged from 0.6%[13] to 5.44%[8] while cost per capita ranged from 85.53 US$ PPP [24]to 1,012.21 US$ PPP[8]. On the other hand, total direct cost as percentage of GDP ranged from 0.08%[25] to 0.81%[8]. When only the 7 studies[7,9,10,14,15,24,25] in which 5 main costs components (health care cost, crime and law enforcement cost, property damage, premature mortality, and reduced productivity) were included, the total cost as % GDP ranged from 0.45% [24] to 2.11% [10] while cost per capita ranged from 85.53 US$ PPP [24]to 850.86 US$ PPP[10].

**Table 7:** Total cost estimates in US$PPP 2007, cost as % of GDP (2007), cost per capita, and total direct cost as % of GDP (PPP) 2007 in the 12 selected countries

Country/ Study Year	Total cost in PPP US$ 2007 (Million)	Cost as % GDP (PPP)2007	Cost per capita (PPP US$ 2007)	Total Direct cost as %GDP
Australia 1998-9 [7]	6,818.6	1.09	359.8	0.5
Canada 2002 [15]	13,406.3	1.24	428.04	0.63
France 1997[9]	22,376	1.44	384.4	0.72
Germany 2002 [12]	30,847.15	1.24	373.77	0.43
Japan 1987 [14]	62,461.8	3.15	511.85	0.62
The Netherlands 2001 [13]	3,314.22	0.6	206.49	0.31
New Zealand 1991 [8]	930.69-3,542.74	1.43-5.44	265.9-1,012.21	0.47-0.81
Portugal 1995 [24]	853.64	0.45	85.53	0.11
Sweden 2002 [11]	2,390-3,441	0.88-1.27	267.38-384.89	0.43-0.47
South Korea 2000 [5]	24,913.7	2.76	530.08	0.77
Thailand 2006 [25]	9,767.7	1.98	149.63	0.08
US 1998 [10]	234,854.2	2.11	850.86	0.58

## Discussion

Despite some agreement on the inclusion or exclusion of particular cost items in the studies, overall the methodologies varied considerably. This makes a direct comparison of results very difficult. In addition, the variation in the estimated cost as a percentage of GDP and cost per inhabitant across countries/studies can also be explained by the factors such as the differences in characteristics of the study population, the drinking prevalence and patterns, and the health care and economic structures of the settings. Furthermore, the number and type of diseases attributable to alcohol included in the estimation is highly influential and varied across studies.

With regards to the cost components in each study, according to WHO guidelines[22] transfer costs should not be included in the estimation, as the transfer of ownership from the payer to the receiver does not affect the amount of resources available to the society. However, the review found that several transfer costs, including disability pensions, accident compensation, and social security payments were incorporated in some studies [8,13,14,26]. The inclusion of the cost of stolen property is somewhat controversial. Generally, this cost can be viewed as involuntary transfer and therefore, should not be included. On the other hand, it was found that the value of stolen property was typically lower than the value it had been before it was stolen. As a result, this value reduction can be considered as actual social costs, and can be incorporated in the estimation. Consistent with this argument, instead of using the original value of the stolen property, two studies [4,11] that incorporated this cost in their analyses used the reduction in value of the stolen property in their estimations.

Concerning the cost of alcoholic beverages themselves, when assuming rational consumer behaviour such private costs are considered to be offset by the benefits from consumption, and hence are not included in the analysis. However, in the case of addictive substances, including alcohol, addictive behaviour seems to violate the assumption of rational consumer behaviour since the addict may derive limited or no utility at all from drinking, yet will continue to do so anyway. According to the recommendation of the WHO guidelines[22], there are two approaches to dealing with this situation. The first approach is to treat these narcotic substances as conventional goods assuming that even dependent users are consuming rationally, and hence the cost of drug consumption is not included in the estimation. The other approach suggests that the proportion of drug consumption judged to be excessive should be estimated and subsequently counted in the estimation. Consistent with the latter approach, three [6,7] out of four studies [5-7] that incorporated the cost of alcoholic beverage as one of the direct costs in their estimation took into account only those costs thought to be addictively consumed, by assuming that 20% of the total consumption had been consumed by addictive drinkers.

The economic cost of alcohol can be useful for policy making only when performed appropriately with minimum biases. These findings clearly depict the need for a set of methodological guidelines for estimating the economic cost of alcohol consumption. Apart from the fact that many of the studies were conducted before the introduction of the WHO guidelines in 2003, the variation across studies is also attributable to several other factors, including the availability and accuracy of data in different countries. Estimating the economic cost of alcohol consumption requires a comprehensive list of data, including population structure, morbidity and mortality, prevalence of specific health problems, unit costs of health care and other related services, and, importantly, the proportion of alcohol attributable to each this. Such data are not always readily accessible, especially in developing countries, where local information is rarely fully available and of sufficient quality. Investment in these countries in data infrastructure is a fundamental necessity to cost estimation for this and other contexts; this review can assist in identifying the most significant cost components for which data are most needed. Besides primary data collection, estimates from external sources such as other countries with similar circumstances can be used, rather than omitting essential components from the estimation altogether.

Lastly, estimates of the economic cost of alcohol may be conducted using different approaches, i.e. prevalence VS incidence approach, and human capital VS demographic approach. As mentioned earlier, different approaches are complementary rather than contradictory. To select which approach to use is depending on the purpose of the study since different approaches are appropriate for different purposes. For example, the prevalence approach may be useful for government budgeting purpose while the incidence approach is more relevant for measuring the impact of alcohol policy. Similarly, human capital approach gives an estimate of the present and future cost due to alcohol-related mortality in a given year while the demographic approach gives the present cost of alcohol-related mortality in past and present year. If possible, the preferred method might be to conduct economic cost study which employed both human capital and demographic approach, and compare the results. Nevertheless, to select the most appropriate approach is also depending on the availability of information, which may vary across settings.

## Conclusion

Notwithstanding the disparities in methodologies and cost components in the individual studies, this review draws some useful conclusions that have attracted a great deal of political and public attention in terms of the economic burden of alcohol consumption worldwide. According to the review, the economic burden of alcohol on society is substantial, accounting for 0.45% to 5.44% of GDP. The findings from this study can be used as economic evidence to support the formulation of alcohol-related policy and to draw public attention to the negative economic impact of alcohol has not only on individual drinkers, but also on society at large. However, given the lack of generalizability across the different settings, such cost estimation should be conducted in a localized manner, especially in developing countries where very few such studies are currently available.

## Competing interests

The authors declare that they have no competing interests.

## Authors' contributions

MT conceived of the study, participated in its design and coordination, carried out the review, and drafted the manuscript. YT conceived of the study, participated in its design and helped to draft the manuscript. JY performed literature search and carried out the review. CL performed literature search and carried out the review. UC conceived of the study, and participated in its design and helped to draft the manuscript. All authors read and approved the final manuscript.
